# Parliament and Publications

**Published:** 1911-03-04

**Authors:** 


### PARLIAMENT AND PUBLICATIONS.

### THE REGISTRAR-GENERAL'S REPORT.

THE seventy-second annual report of the Registrar-General of Births, Marriages, and Deaths in England and Wales dealing with the year 1909 has just been published. A preliminary survey of the report shows that the birth rate in 1909 was the lowest on record up to that date, but the trend is still downwards, the provisional rate for 1910 being yet lower, viz., 24.8 per 1,000. The births registered in 1909 numbered 914,472, being equal to a rate of 25.6 per 1,000 living. In the year 1876 the birth-rate in this country attained the higest point on record—namely, 36.3 per 1,000 living, since which date the ratio has, with a few insignificant exceptions, fallen year by year. The death rate in 1909 was also the lowest on record up to that date, but the provisional rate for 1910 is no less than 1.1 per 1,000 lower. The deaths of 518,003 persons were registered in England and Wales during the year 1909, corresponding to a rate of 14.5 per 1,000 of the estimated population. The annual rate of mortality reached its highest record point in the quinquennium 1846-50, when it stood at 22.4 per 1,000 persons living. From that time it fell gradually and intermittently till 1891-95, when it stood at 18.5, but since then the fall has been steadier and much more rapid, the total decline since recorded being almost equal to that exhibited during the preceding 45 years. The death rate in 1909 was 65 per cent. only of that recorded in the later "forties." The rate of infantile mortality in England and Wales was 109 per 1,000 births in 1909. This is by far the lowest recorded up to that time, but the provisional figures show that the rate for 1910 will be lower still. The death rate in 1909 from cancer was the highest on record, showing an increase of as much as 29 per million living upon the rate in the preceding year, the highest recorded till then. The death rate from tuberculosis as a whole and from phthisis were, on the other hand, the lowest on record. The death rates from whooping-cough and enteric fever were also the lowest on record, while that attributed to diphtheria was the lowest since 1881, if not lower than that of any previous year for which the figures are available. The marriage rate was the lowest since 1888, the marriages last year corresponding to a rate of 14.6 persons married per 1,000 of the population at all ages, or 0.3 per 1,000 below the rate in 1908.—"Registrar-General's Report," Cd. 5485, price 2s. 6d.

### VACCINATION IN THE NAVY.

As the danger of the spread of infection is very largely increased by the peculiar conditions of life on board ship, it is not proposed to relax the present regulations with regard to vaccination. This statement was made by Mr. McKenna in reply to Mr. Pointer, who asked whether, in view of the fact that the number of smallpox cases in the Navy had been very small for the last few years, he could see his way to extend to naval recruits the same liberty of conscience in regard to vaccination as had already been granted to other branches of the Government services.

### SLEEPING-SICKNESS IN RHODESIA.

IN reply to a question, Mr. Harcourt stated that considerable areas in the Luapula and Luangwas valleys had been declared sleeping sickness areas. The British South Africa Company has strengthened its medical staff, and it is now sending out a special commission of investigation with a scientific staff and a fully equipped laboratory.

### A SICK-LEAVE CERTIFICATE.

MR. DELANY asked the Secretary to the Treasury whether he was aware that the Board of Works on a recent occasion refused to accept the certificate of Sir Christopher Nixon in the case of an application for sick-leave made by a clerk; and could he state on what grounds the certificate of a former President of the Royal College of Physicians was passed over and the clerk ordered to consult Dr. Wynn. In his reply Mr. Hobhouse stated that the facts were not correct. The clerk consulted Sir Christopher after he had been examined by Dr. Wynn, and not before. The Board did not refuse to accept Sir Christopher's certificate. They acted on it after personal communication with him.

### REBATE OF THE MOTOR-SPIRIT DUTY.

MEDICAL practitioners claiming rebate of the duty on motor-spirit are not required to prove that manufacturers of and dealers in motor-spirit have paid the duty. This is the effect of a reply given by Mr. Hobhouse to a question asked in the House of Commons.

### EXPERIMENTS ON DOGS.

THE Bill to prohibit experiments upon dogs which has been introduced in the House of Commons by Sir Frederick Banbury has now been printed. It seeks to make it unlawful "to perform any experiment of a nature likely to cause pain or disease to any dog for any purpose whatsoever, either with or without anæsthetics, and no person or place shall be licensed for the purpose of performing any such experiments." The penalty for the first offence is a sum not exceeding £10, for a second £50, and for a third £100.

### COMPOSITION OF SECRET REMEDIES.

THE Home Secretary has informed Mr. Stephen Collins that he is making inquiries into the question of the sale of patent medicines with a view to determining whether it is desirable to appoint a Select Committee to consider the advisability of making it compulsory upon all manufacturers of medicines liable to stamp duty to disclose the composition of such medicines on the labels.

### SUGGESTED STANDARD FOR BREAD.

THE Foods Department of the Local Government Board is investigating the question of the comparative nutritive values of bread made from various classes of flour. The War Office is also considering the question of the supply of standard bread. These facts were stated in reply to questions by Mr. Goldman, who drew attention to the "opinions of eminent medical men as to the national importance of establishing a standard for bread containing a minimum of 80 per cent. of the nutritive value of wheat."

### THE MIDWIVES BILL.

MR. JOHN BURNS has agreed to receive a deputation from the Municipal Corporations Association on Clause 17 of the Midwives Bill which was introduced into the House of Commons last year and may be re-introduced this session. Clause 17 provides that "Where a duly qualified medical practitioner has been summoned upon the advice of a certified midwife attending a woman in child-birth to render assistance in a case of emergency in pursuance of any rule framed by the Central Midwives Board, he shall, on complying with the prescribed conditions, be entitled to recover from the Board of Guardians of the Poor Law Union in which the woman resided such fee in respect of his attendance as may be prescribed."

